# Endoscopic Treatment of Pilonidal Sinus Disease: State of Art and Review of the Literature

**DOI:** 10.3389/fsurg.2021.812128

**Published:** 2022-01-04

**Authors:** Michele Manigrasso, Pietro Anoldo, Grazia Cantore, Alessia Chini, Anna D'Amore, Nicola Gennarelli, Francesco Maione, Alessandra Marello, Pietro Schettino, Carmen Sorrentino, Sara Vertaldi, Loredana Maria Sosa Fernandez, Giovanni Domenico De Palma, Marco Milone

**Affiliations:** ^1^Department of Advanced Biomedical Sciences, “Federico II” University of Naples, Naples, Italy; ^2^Department of Clinical Medicine and Surgery, “Federico II” University of Naples, Naples, Italy; ^3^EMBRYOS Fertility Center and Day Surgery, Palazzo Colosseum, Battipaglia, Italy

**Keywords:** pilonidal, endoscopic, VAAPS, EPSiT, PEPSiT, state of art, review

## Abstract

**Background:** Pilonidal sinus disease (PSD) is a chronic troublesome pathology of the natal cleft of the sacrococcygeal region, with an estimated incidence of 26 cases in every 100,000 inhabitants. The aim of this review is to give a snapshot of the current literature on the endoscopic approach to PSD.

**Methods:** A search on endoscopic treatment of pilonidal disease was performed according to PRISMA guidelines, adopting the following search terms: (pilonidal OR sacrococcygeal) and (endoscopic OR VAAPS OR EPSiT OR minimally invasive OR video-assisted OR video assisted).

**Results:** Thirty-four articles were included in the final analysis, among which 23 were on adults and 11 were on pediatric population. The endoscopic approach is associated with painless postoperative pain, good aesthetic results, short time off work, and high patient satisfaction.

Despite these advantages in short-term outcomes, results on recurrence rate in a long-term follow up are needed to definitively confirm the importance of this technique.

**Conclusions:** The endoscopic approach is associated with significant postoperative advantages over other standard surgical approaches, and it should be included in the surgical portfolio for the treatment of PSD. According to the Italian guidelines, this technique could be considered as the gold standard for limited PSD. However, the favorable short-term-outcomes and lack of reliable data on long-term follow-up must be a stimulus to perform further high-quality studies to give definitive conclusions on this technique.

## Introduction

Pilonidal sinus disease (PSD) is a chronic troublesome pathology of the natal cleft of the sacrococcygeal region, with an estimated incidence of 26 cases in every 100,000 inhabitants (1). It afflicts mainly males aged between 15 and 30 years (2).

In the past decades, several approaches have been proposed to treat this common disease, but a gold standard treatment has still not been recognized, especially in cases of complex PSD (3–7).

The minimally invasive endoscopic approach, first described by both Meinero et al. (8) and Milone et al. (9) independently has completely revolutionized the surgical approach to PSD.

In fact, since its introduction, this approach has gained momentum among surgeons because of relatively absent postoperative pain, good aesthetic results, low infection rate, and low long-term recurrence rate (10–16).

The aim of this review is to give a snapshot of the current literature on the endoscopic approach to PSD.

## Materials and Methods

To identify all available studies, a detailed search on endoscopic treatment of pilonidal sinus disease was performed according to the PRISMA (Preferred Reporting Items for Systematic reviews and Meta-Analyses) guidelines (17). A systematic search was conducted through electronic databases (PubMed, Web of Science, Scopus, EMBASE), adopting the following search terms: (pilonidal OR sacrococcygeal) and (endoscopic OR VAAPS OR EPSiT OR minimally invasive OR video-assisted OR video assisted). All articles that have been published since the introduction of this new technique were included. The literature search and review of the articles were performed by two independent reviewers and the last search was performed on October 30, 2021. In addition, reference lists of all the retrieved articles were manually reviewed. Studies involving animals, systematic reviews, meta-analyses, congress abstracts, and non-English articles were excluded. Meta-analyses and systematic reviews were analyzed to obtain further studies.

## Results

**Figure 1:**
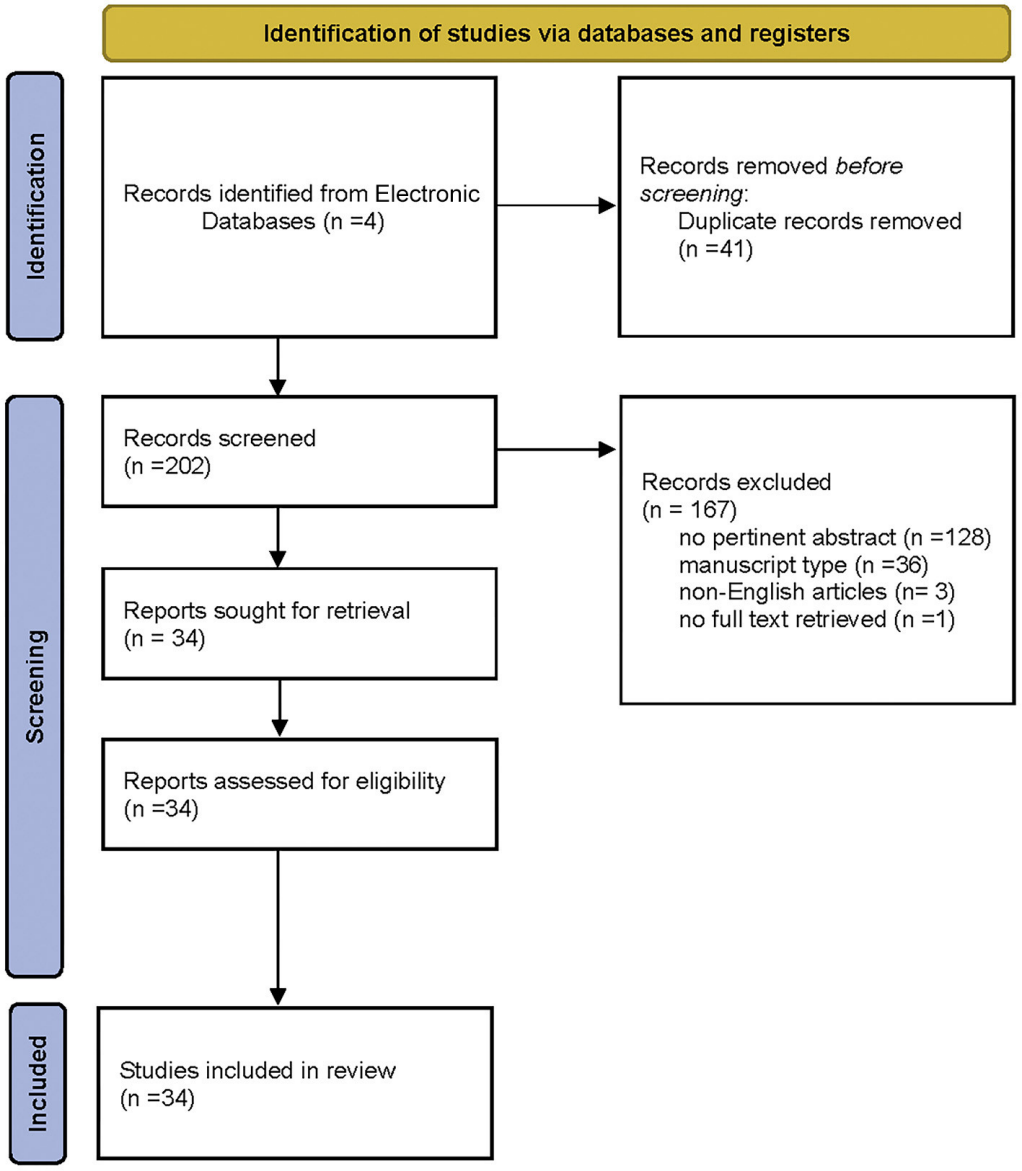
PRISMA 2020 flow diagram for new systematic reviews which included searches of databases and registers only. From: (46).

A total of 243 studies were obtained from the literature search. After removing all duplicates, 202 articles were reviewed. Of these, 168 articles were excluded for several reasons: 128 because of not pertinent title/abstract, 36 for manuscript typology (21 reviews/meta-analyses, 8 letters to the editor/commentary, 8 case reports/technical notes), 3 because they were non-English articles, and 1 because no full text was retrieved. Thus, 34 articles were included in the final analysis (8–11, 13, 14, 18–45), among which 23 were on adults and 11 were on pediatric population. The exclusion criteria are represented in Figure 1 (PRISMA flowchart). Cohort and comparative studies on adult population are summarized in Tables 1, 2, respectively, while cohort and comparative studies on the pediatric population are summarized in Tables 3, 4, respectively.

**Table 1:** Cohort studies on the endoscopic approach in the adult population.

**References**	**Title**	**Journal**	**Type**	**Comparative**	**Type of sinus (primary/recurrent/acute)**	**Patients number**	**Endoscopic technique**	**Other technique**	**Infection**	**Recurrence**	**Follow-up (months)**
Meinero et al. (8)	Endoscopic pilonidal sinus treatment (E.P.Si.T.)	Techniques in Coloproctology	Prospective	No	Recurrent (8)	11	EPSiT		0	0	9
Milone et al. (9)	Video-assisted ablation of pilonidal sinus: A new minimally invasive treatment—A pilot study	Surgery	Prospective	No		27	VAAPS		0	1	12
Chia et al. (42)	Endoscopic Pilonidal Sinus Treatment in the Asian Population	Surgical Laparoscopy Endoscopy & Percutaneous Techniques	Retrospevtive	No	Recurrent (2)	9	EPSiT			1	2.5
Meinero et al. (11)	Endoscopic pilonidal sinus treatment: a prospective multicentre trial	Colorectal Disease	Prospective	No	Recurrent	250	EPSiT		NR	13	NR
Giarratano et al. (13)	Endoscopic Pilonidal Sinus Treatment: Long-Term Results of a Prospective Series	Journal of the Society of Laparoendoscopic Surgeons	Prospective	No	Recurrent (9)	77	EPSiT		0	6	25
Gecim et al. (44)	Endoscopic Pilonidal Sinus Treatment Combined With Crystalized Phenol Application May Prevent Recurrence	Diseases of the Colon & Rectum	Prospective	No	Recurrent (1)	23	EPSiT		0	0	24
Jain et al. (45)	Endoscopic pilonidal abscess treatment: a novel approach for the treatment of pilonidal abscess	Annals of Royal College of Surgeon	Retrospective	No	Acute and Recurrent (9)	19	EPAT		NR	0	10.5
Meinero et al. (19)	Endoscopic pilonidal sinus treatment (EPSiT) in recurrent pilonidal disease: a prospective international multicenter study	International Journal of Colorectal Disease	Prospective	No	Recurrent	122	EPSiT		NR	6	16
Mendes et al. (20)	Brazilian and argentinean multicentric study in the surgical minimally invasive treatment of pilonidal cyst	ABCD Arq Bras Cir Dig	Prospective	No		67	EPSiT		0	6	NR
Kalaiselvan et al. (21)	Short-term outcomes of endoscopic pilonidal sinus treatment	Annals of Royal College of Surgeon	Retrospective	No	Recurrent (35)	74	EPSiT		0	0	13
Khafagy et al. (22)	The endoscopic treatment of pilonidal sinus disease: a short-term case-series study	Annals of Saudi Medicine	Retrospective	No	Recurrent (14)	35	EPSiT		0	2	6
Eastment et al. (24)	Outcomes of minimally invasive endoscopic pilonidal sinus surgery	Asian Journal of Endoscopic Surgery	Retrospective	No	Acute (1) Recurrent (4)	9	EPSiT		0	3	28
Manigrasso et al. (25)	Early versus delayed endoscopic treatment of acute pilonidal abscess: a propensity score-matched analysis	International Journal of Colorectal Disease	Prospective	No	Acute	82	VAAPS		4	5	60
Azhough et al. (26)	Endoscopic pilonidal sinus treatment: A minimally invasive surgical technique	Asian Journal of Endoscopic Surgery	Retrospective	No		100	EPSiT		0	4	14.35 ± 2.42
Manigrasso et al. (27)	Endoscopic Approach to Recurrent Pilonidal Sinus: A Retrospective Analysis	Journal Of Laparoendoscopic & Advanced Surgical Techniques	Retrospective	No	Recurrent	63	VAAPS		5	3/63 at 1 year 4/34 at 3 years 3/13 at 5 years	60
Gallo et al. (28)	Endoscopic Pilonidal Sinus Treatment: A Tertiary Care Academic Center Experience	Frontiers in Surgery	Retrospective	No	Recurrent (4)	32	EPSiT		NR	NR	22 (4–42) ± 11.49
Foti et al. (29)	A minimally invasive approach to pilonidal disease with endoscopic pilonidal sinus treatment (EPSiT): a single-center case series with long-term results	Techniques in Coloproctology	Retrospective	No	Primary (22) Recurrent (24)	46	EPSiT		2	5	62
Baxter et al. (30)	The EPIC procedure (Endoscopic-assisted Pilonidal Irrigation and Cleaning): a simple and effective treatment for pilonidal disease	Surgical Endoscopy	Retrospective	No	Primary	20	EPIC		1	3	27.95

**Table 2:** Comparative studies on the endoscopic approach in the adult population.

**References**	**Title**	**Journal**	**Type**	**Comparative**	**Type of sinus (primary/recurrent/acute)**	**Patients number**	**Endoscopic technique**	**Other technique**	**Infection**	**Recurrence**	**Follow-up (months)**
Javed et al. (43)	Comparison of conventional incision and drainage for pilonidal abscess versus novel endoscopic pilonidal abscess treatment (EPAT)	Techniques in Coloproctology	Retrospective	Yes	Acute	40	EPAT (20)	Conventianal treatment (20)	NR	0 (EPAT) 3 (convention al treatment)	3–6
Milone et al. (10)	Safety and Efficacy of Minimally Invasive Video-Assisted Ablation of Pilonidal Sinus A Randomized Clinical Trial	JAMA surgery	RCT	Yes	Chronic non-recurrent	145	VAAPS (76)	Bascom cleft lift (69)	1 (VAAPS) 5 (Bascom cleft lift)	3 (VAAPS) 4 (Bascom cleft lift)	NR
Milone et al. (18)	Video-assisted ablation of pilonidal sinus (VAAPS) versus sinusectomy for treatment of chronic pilonidal sinus disease: a comparative study	Updates in Surgery	Prospective	Yes	Chronic non-recurrent	80	VAAPS (40)	Sinusectomy (40)	5 (VAAPS) 12 (sinusectomy)	3 (VAAPS) 10 (sinusectomy)	60
Romaniszyn et al. (23)	Long-term results of endoscopic pilonidal sinus treatment vs. Limberg flap for treatment of difficult cases of complicated pilonidal disease: a prospective, nonrandomized study	Colorectal Disease	Prospective	Yes	Recurrent	60	EPSiT (26)	Limberg (34)	3 (EPSiT) 3 (Limberg)	13 (EPSiT) 11 (Limberg)	27
Milone et al. (14)	Long-term results of a randomized clinical trial comparing endoscopic versus conventional treatment of pilonidal sinus	International Journal of Surgery	RCT	Yes	Chronic non-recurrent	145	VAAPS (74)	Bascom cleft lift treatment (67)	NR	18 VAAPS 16 Bascom cleft lift	60

**Table 3:** Cohort studies on endoscopic treatment in the pediatric population.

**References**	**Title**	**Journal**	**Type**	**Comparative**	**Type of sinus (primary/recurrent/acute)**	**Patients number**	**Endoscopic technique**	**Other technique**	**Infection**	**Recurrence**	**Follow-up (months)**
Esposito et al. (31)	Pediatric Endoscopic Pilonidal Sinus Treatment, a Revolutionary Technique to Adopt in Children with Pilonidal Sinus Fistulas: Our Preliminary Experience	Journal Of Laparoendoscopic & Advanced Surgical Techniques	Retrospective	No		15	PEPSiT		0	0	6
Pini Prato et al. (32)	Preliminary report on endoscopic pilonidal sinus treatment in children: results of a multicentric series	Pediatric Surgery International	Retrospective	No	Recurrent (6)	43	PEPSiT		NR	5	4
Esposito et al. (34)	Pediatric Endoscopic Pilonidal Sinus Treatment: An Effective Procedure for Children with Recurrent Pilonidal Sinus Disease After Failed Open Surgery	Journal of Laparoendoscopic & Advanced Surgical Techniques	Retrospective	No	Recurrent (10)	40	PEPSiT		0	0	18
Esposito et al. (35)	Pediatric Endoscopic Pilonidal Sinus Treatment (PEPSiT) in Children With Pilonidal Sinus Disease: Tips and Tricks and New Structurated Protocol	Frontiers in Pediatrics	Retrospective	No	Recurrent (15)	127	PEPSiT		0	6	18
Esposito et al. (36)	Technical standardization of MIS management of children with pilonidal sinus disease using pediatric endoscopic pilonidal sinus treatment (PEPSiT) and laser epilation	Journal of Pediatric Surgery	Retrospective	No	Recurrent (10)	59	PEPSiT		0	1	18
Esposito et al. (37)	Standardization of Pre- and Postoperative Management Using Laser Epilation and Oxygen-Enriched Oil-Based Gel Dressing in Pediatric Patients Undergoing Pediatric Endoscopic Pilonidal Sinus Treatment (PEPSiT)	Lasers in Surgery and Medicine	Retrospective	No	Recurrent (10)	87	PEPSiT		5	7	NR
Gökbuget et al. (38)	Endoscopic pilonidal sinus treatment (EPSiT) in the pediatric age group: Short-term results	Ulusal Travma ve Acil Cerrahi Dergisi	Retrospective	No	Recurrent (8)	29	EPSiT		NR	8	8.3 ± 3.34
Dotlacil et al. (40)	Initial experience with minimally invasive treatment of pilonidal sinus in children	Videosurgery and Other Miniinvasive Techniques	Retrospective	No		17	PEPSiT		0	2	10
Esposito et al. (41)	Pediatric endoscopic pilonidal sinus treatment (PEPSiT): what we learned after a 3-year experience in the pediatric population	Updates in Surgery	Retrospective	No	Recurrent (15)	152	PEPSiT		8	7	12.8

**Table 4:** Comparative studies on endoscopic treatment in the pediatric population.

**References**	**Title**	**Journal**	**Type**	**Comparative**	**Type of sinus (primary/recurrent/acute)**	**Patients number**	**Endoscopic technique**	**Other technique**	**Infection**	**Recurrence**	**Follow-up (months)**
Sequeira et al. (33)	Endoscopic pilonidal sinus treatment versus total excision with primary closure for sacrococcygeal pilonidal sinus disease in the pediatric population	Journal of Pediatric Surgery	Retrospective	Yes		84	PEPSiT (21)	Excision followed by primary closure (EPC) (63)	1 (PEPSiT) 12 (EPC)	2 (PEPSiT) 13 (EPC)	11.9 (PEPSiT) 24.7 (EPC)
Pérez-Bertólez et al. (39)	Advantages of endoscopic pilonidal sinus treatment	Cirurgia Pediatrica	Prospective	Yes		49	PEPSiT (14)	Excision and healing by secondary intention (EHSI) (23), excision and primary closure (EPC) (12)	3 (EHSI) 2 (EPC) 0 (PEPSiT)	0	14.8

## Discussion

In the past decades, several surgical treatment have been proposed to approach PSD, although the ideal treatment still remains controversial (3–5, 47). In the last decade, a new minimally invasive technique was added to the surgical portfolio: the endoscopic approach (8, 9).

Born independently by the ideas of Meinero et al. (8) and Milone et al. (9), the techniques ARE based on the ablation under direct vision of the pilonidal cavity by the adoption of an endoscope through the lower pit of the sinus, without any other skin incision. The advantage of this technique over the standard surgical excision of the sinus cavity could derive by the possibility to exactly define the involved area and to completely remove the hair and sinus cavity under direct vision.

Since the introduction of the techniques, EPSiT and VAAPS, respectively, the interest for this new approach has gained momentum among the surgeon and several studies have been published (10, 13, 19–22, 24–30, 42, 44).

However, most of these were non-comparative, retrospective studies and with a relatively small population. To compare the clinical results of endoscopic approach to PSD with those of traditional surgical treatments, two comparative studies (18, 23) and one RCT were published (10).

In a comparison between sinusectomy and the endoscopic approach by Milone et al. (18), the recurrence rate was significantly lower in the endoscopic group (7.5 vs. 25%) after a mean 4-year follow-up, and a trend toward reduction in the endoscopic group in terms of infection rate was retrieved.

At difference, the comparison by Romaniszyn et al. (23) between endoscopic treatment and Limberg flap for treatment of difficult cases of complicated pilonidal disease showed that the endoscopic procedure had a significantly lower success rate than the Limberg flap procedure, but a lower risk of postoperative complications.

The feasibility and safety of the endoscopic approach have been assessed by Milone et al. in the only one randomized comparison present in the literature between VAAPS and Bascom cleft lift (10), demonstrating that VAAPS implied a shorter time off work and lower postoperative infection rate and postoperative pain. Long-term results of RCT (14) demonstrated a similar recurrence rate between two groups in a 5-year follow-up, but in a small group of patients.

The feasibility of the endoscopic surgery for chronic and recurrent pilonidal disease has encouraged surgeon in its adoption even in the acute pilonidal abscess (25, 43, 45). For this reason, the endoscopic pilonidal abscess treatment (EPAT) was introduced by Javed et al. (43).

Since its introduction, two comparative studies have been performed over the last years (25, 43), demonstrating that the endoscopic approach to acute pilonidal sinus was associated with reduced postoperative pain and lower duration of wound healing and time off work, with no differences in terms of definitive surgery needed.

The adoption of the endoscopic technique has gained large consensus even among pediatric surgeons since the first trial was promoted by Esposito et al. on a pediatric population (31).

Even in pediatric populations, this technique seemed to be associated with low postoperative pain, short length of hospital stay (about 1 day), and better outcomes than open technique.

Several non-comparative reports have demonstrated similar results, with a recurrence rate ranging from 0 to about 6% (32, 34–38, 40, 41). In this setting, several authors considered the PEPSiT as the gold standard of care in cases of PSD in the pediatric population.

The analysis of the current literature seems completely in favor of the endoscopic approach for both the pediatric and adult populations.

In all the published studies, this technique seems to be associated with good postoperative results, and compared with other surgical approaches, the endoscopic technique seems to be associated with better postoperative outcomes and high patient satisfaction.

Furthermore, according to the latest Italian guidelines (5), the endoscopic approach could be considered as the gold standard for cases of limited pilonidal sinus disease.

However, some important limitations of the current literature should be addressed.

First, the published trials are mostly retrospective, with a relatively small population. Furthermore, the most important blind spot of this technique remains to be the long-term follow up. According to Dettmeret al. (48), the litmus test of every technique is the long-term follow-up, and in the case of pilonidal surgery it should be at least 5 years, as demonstrated by Doll and others (49, 50).

In this setting, the shorter follow-up of the included studies remains an important bias to give definitive conclusions on this technique. Nevertheless, it is important to underline that even in the case of other surgical approaches to PSD, the results on long-term follow-up longer than 5 years are still lacking, as reported by the meta-analysis by Milone et al. (51).

Other open issues of the current literature should be addressed. In fact, no data are present about the learning curve and the real cost of this technique. About the first aspect, it would be interesting to perform studies on the learning curve among young surgeons or residents to assess how many interventions are needed to gain adequate proficiency.

For what concerns the second aspect, it is rational to think that the cost of the endoscopic approach could be higher than that of traditional techniques, which just require the use of a scalpel, monopolar electrocautery, and suture threads. However, considering better results in terms of postoperative recovery and shorter time off work, the economic side could be balanced by the high patient satisfaction.

Despite these limitations, we can conclude that the endoscopic approach is associated with significant postoperative advantages over other standard surgical approaches, and that it should be included in the surgical portfolio for the treatment of PSD. However, the favorable short-term-outcomes and lack of reliable data on long-term follow up must be a stimulus to perform further high-quality studies to give definitive conclusions on this technique.

## Data Availability Statement

The original contributions presented in the study are included in the article/supplementary material, further inquiries can be directed to the corresponding author/s.

## Author Contributions

MMa: conception, design, interpretation of the data, and drafting of the article. MMa, PA, GC, AC, AD'A, NG, FM, AM, PS, CS, SV, and LS: acquisition, analysis, and interpretation of the data. GD and MMi: interpretation of the data, critical revisions, and final approval. All authors contributed to the article and approved the submitted version.

## Conflict of Interest

The authors declare that the research was conducted in the absence of any commercial or financial relationships that could be construed as a potential conflict of interest.

## Publisher's Note

All claims expressed in this article are solely those of the authors and do not necessarily represent those of their affiliated organizations, or those of the publisher, the editors and the reviewers. Any product that may be evaluated in this article, or claim that may be made by its manufacturer, is not guaranteed or endorsed by the publisher.
